# Supplementary figures and images for: The MIR-NAT MAPT-AS1 does not regulate Tau expression in human neurons
Source: PLoS One. 2025 Jan 6;20(1):e0314973. doi: 10.1371/journal.pone.0314973 (PMC11703057; doi:10.1371/journal.pone.0314973)

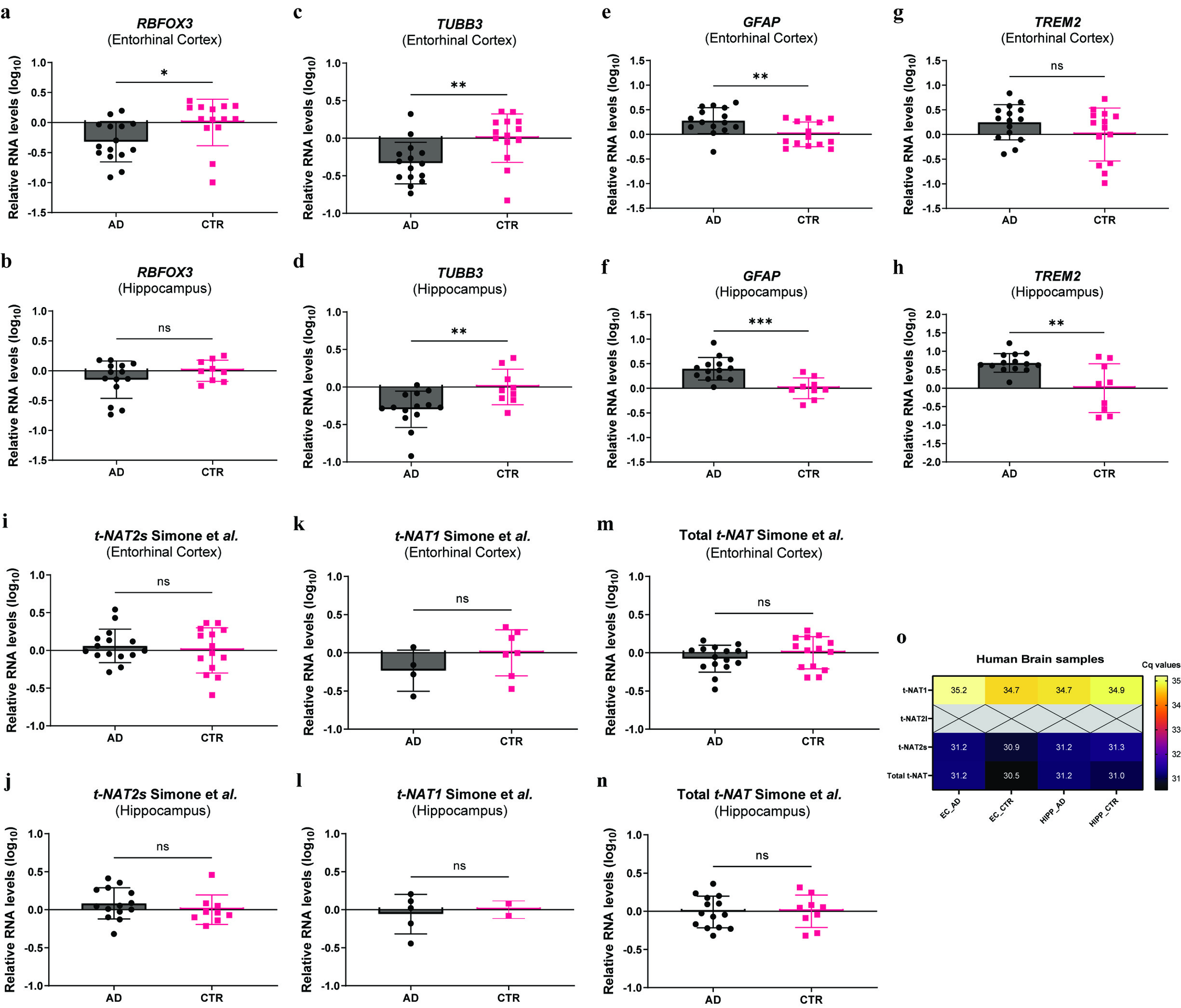

Supplement: S1 Fig — a-h, mRNA expression levels of neuronal markers RBFOX3 (a, b) and TUBB3 (c, d); and reactive astrocytic and microglial markers GFAP (e, f) and TREM2 (g, h), respectively, in human brain samples from AD patients and Control individuals evaluated by RT-qPCR; n = 15 AD and 14 CTR for entorhinal cortex; n = 14 AD and 9 CTR for hippocampus; relative RNA level values are normalized to 2 endogenous control genes and calibrated to Control (CTR) group; data are mean ± SD; Mann-Whitney test (a,b) or unpaired t-test (c-n) with df = 27 for entorhinal cortex and df = 21 for hippocampus, two-tailed p-value (*, p ≤ 0.05; **, p ≤ 0.001). i-n, Expression levels of t-NAT transcripts (i-n) in human brain samples from AD patients and Control individuals evaluated by RT-qPCR; n = 15 AD and 14 CTR for entorhinal cortex; n = 14 AD and 9 CTR for hippocampus; relative RNA level values are normalized to 2 endogenous control genes and calibrated to Control (CTR) group; data are mean ± SD; datapoints for t-NAT1 are missing due to undetectable levels of this transcript in some individuals. Mann-Whitney test or unpaired t-test with df = 27 for entorhinal cortex and df = 21 for hippocampus, two-tailed p value (*, p ≤ 0.05; **, p ≤ 0.001). o, Cq values from t-NAT transcripts in human brain samples were obtained with RT-qPCR. Crossed samples correspond to no amplification. Data are mean. (TIF) [file pone.0314973.s001.tif]

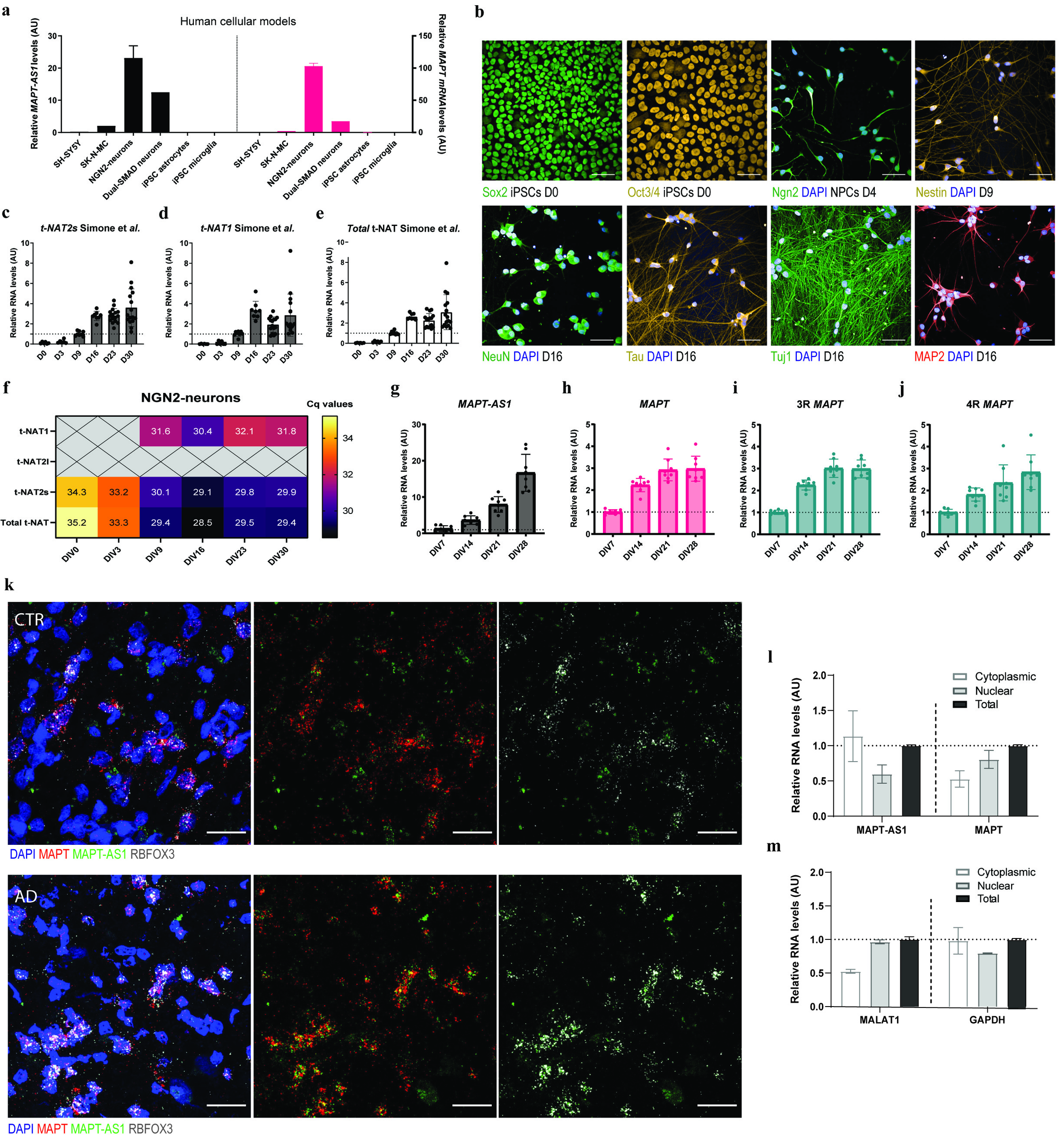

Supplement: S2 Fig — a, Expression levels of MAPT-AS1 and MAPT mRNA in SH-SY5Y and SK-N-MC cells, and human iPSC derived models were evaluated by RT-qPCR; n = 1–2 samples per cell type; astrocytes and microglia analysed at day 126 and day 14, respectively; NGN2-neurons or human iPSCs differentiated to cortical neurons using the Dual-SMAD at days 30 and 28 in vitro, respectively; relative RNA level values are normalized to 2 endogenous control genes and calibrated to average; data are mean (± SD, when applicable). b, Human iPSCs show expression of stem cell markers Sox2 and Oct3/4 at day 0, and NGN2 after treatment with doxycycline for 4 days. Immature neurons at day 9 displayed a neuron-like morphology accompanied by expression of Nestin. Neuronal maturation markers MAP2, Tau, TUJ1 and NeuN are expressed at day 16. DAPI staining was used to detect cell nuclei. Scale bar 50μm; n = 4 independent differentiations of NGN2-neurons. c-e, Expression levels of t-NAT transcripts during differentiation of NGN2-neurons at days 0, 3, 9, 16, 23 and 30 evaluated by RT-qPCR; n = 2 to 6 independent differentiations of NGN2-neurons; relative RNA level values are normalized to 2 endogenous control genes and calibrated to data from 9 days in vitro; data are mean ± SD. f, Cq values from t-NAT transcripts in NGN2-neurons samples were obtained with RT-qPCR. Crossed samples correspond to no amplification. Data are mean. g-h, Expression levels of MAPT-AS1 (g) and MAPT mRNA transcripts (h-j) during differentiation of Dual-SMAD neurons at days 7, 14, 21 and 28 evaluated by RT-sqPCR; n = 8 independent wells per timepoint from 1 differentiation of Dual-SMAD neurons; relative RNA level values are normalized to 2 endogenous control genes and calibrated to data from 7 days in vitro. Data are mean ± SD. k, In situ hybridization showing MAPT (red), MAPT-AS1 (green) and RBFOX3 (white) expression in post-mortem brains from CTR and AD patients. Representative images from 1 CTR and 1 AD patient. Scale bar 30μm. l, m, Expre [file pone.0314973.s002.tif]

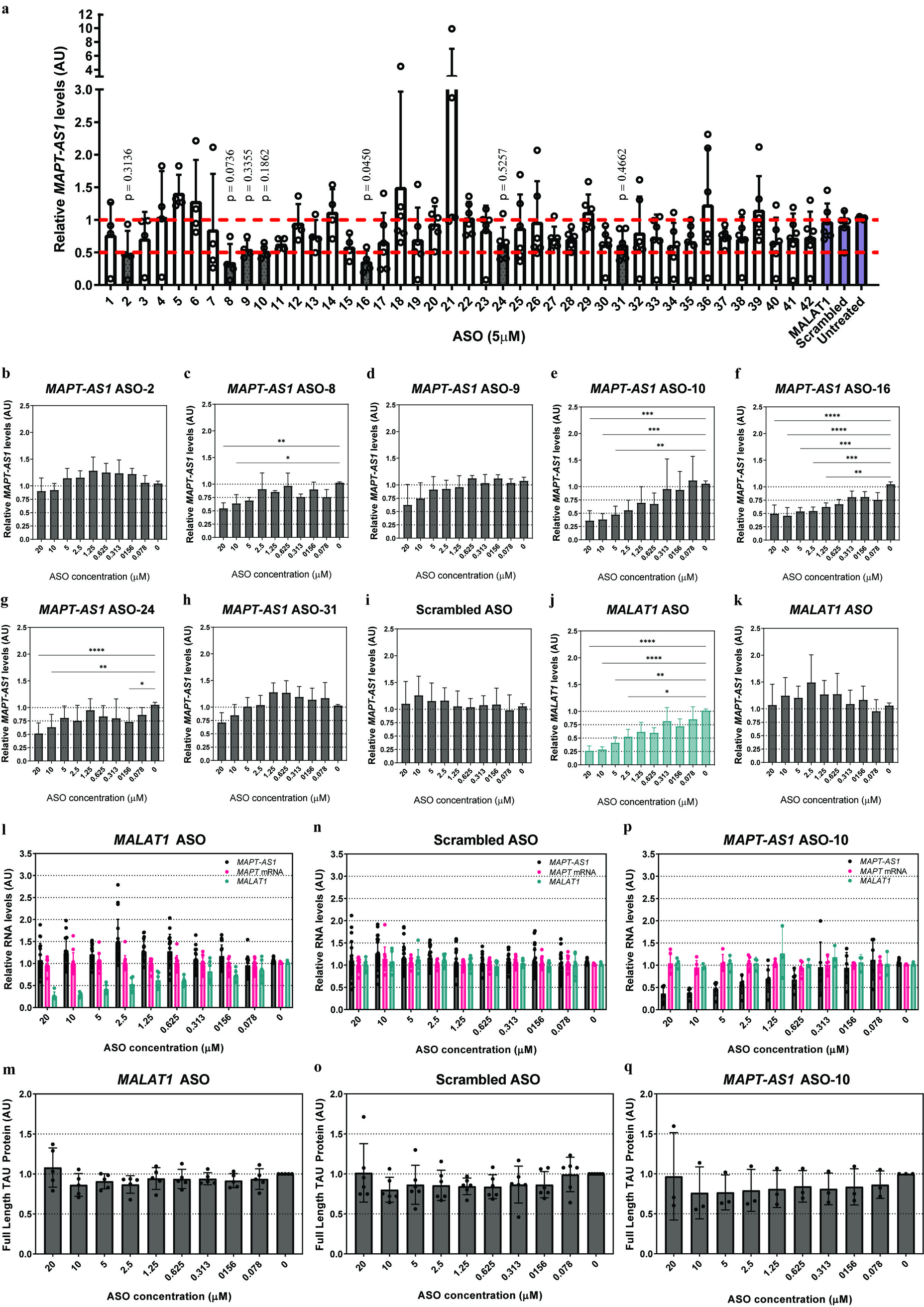

Supplement: S3 Fig — a, SK-N-MC cells were treated with 42 ASOs spanning the entire MAPT-AS1 transcript at a single dose (5 μM) and harvested after 48 hours for RNA analysis; n = 2 independent screening experiments; MAPT-AS1 RNA expression levels were evaluated by RT-sqPCR using 3 independent assays; relative RNA level values are normalized to 2 endogenous control genes and calibrated to untreated condition; Kruskal-Wallis test with Dunn’s multiple comparisons test; data are mean ± SD. b-k, SK-N-MC cells were treated with serially diluted (20 to 0.078μM) lead candidate MAPT-AS1 ASOs (b-h, ASO-2, -8, -9, -10, -16, -21, -24), a MALAT1 ASO (i, j) or a non-targeting ASO (k) and harvested after 72 hours for RNA analysis. l-q, SK-N-MC cells were treated with a MALAT1 ASO (l, m), a non-targeting ASO (n, o), or the lead MAPT-AS1 ASO (p, q) and harvested after 72 hours for RNA and Tau protein analysis. n = 3–4 independent experiments per ASO; RNA expression levels were evaluated by RT-qPCR; relative RNA level values are normalized to 2 endogenous control genes and calibrated to untreated condition. Tau protein levels were assessed using a full-length Tau protein MSD assay; values scaled to untreated condition (average set to 1); all data are mean ± SD. (TIF) [file pone.0314973.s003.tif]

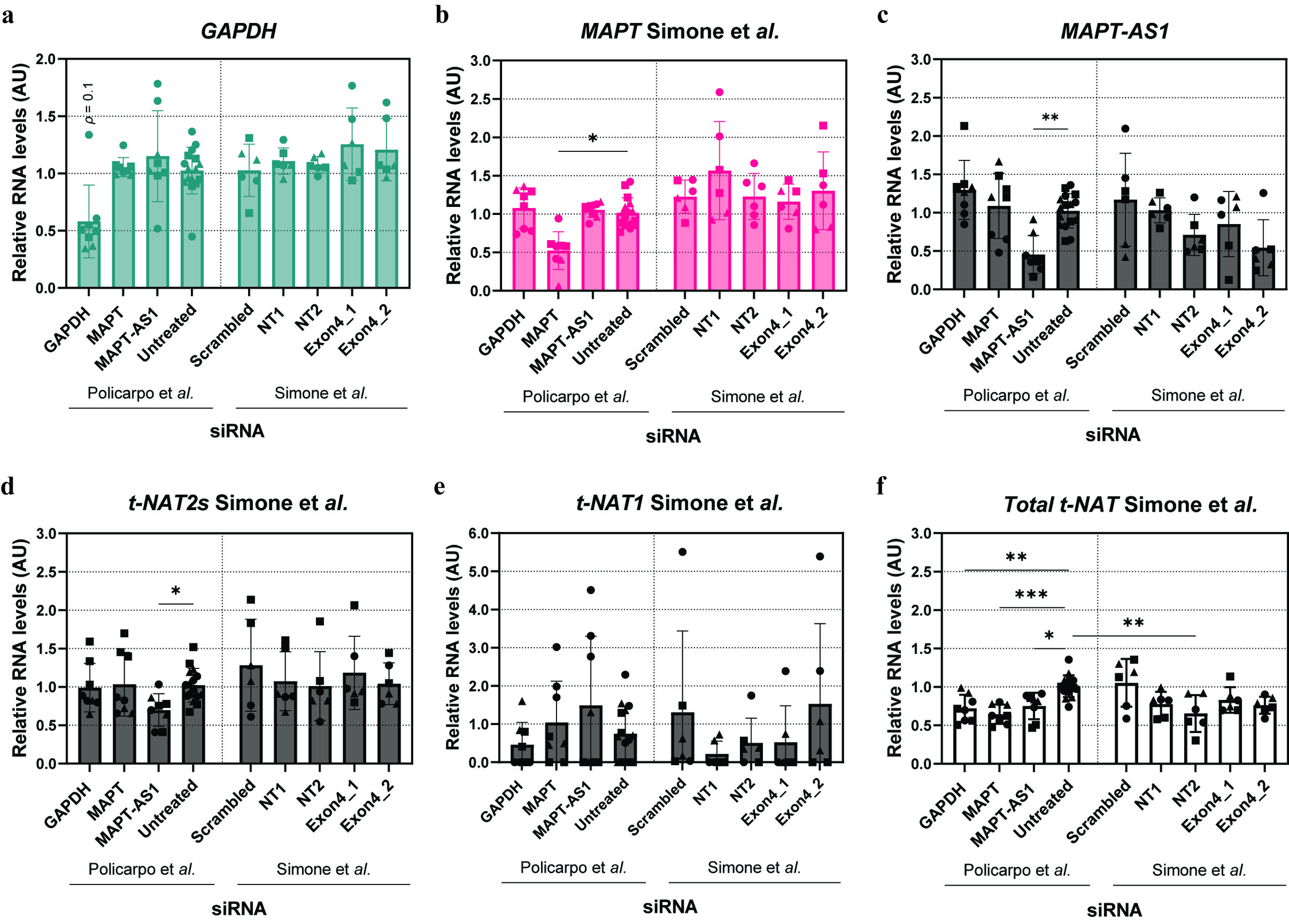

Supplement: S4 Fig — a-e, SH-SY5Y cells were treated with different siRNAs and harvested 48 hours later for RNA analysis. Results from siRNA sequences obtained from Simone et al. [29] are shown on the right panel of each graph, as mentioned. Expression levels of GAPDH (a) and MAPT mRNA (b), MAPT-AS1 (c) and t-NAT transcripts (d-f) were evaluated by RT-qPCR; n = 3 independent experiments per siRNA; data from different experiments indicated as circles, squares or triangles, respectively; relative RNA level values are normalized to 2 endogenous control genes and calibrated to untreated condition; data are mean ± SD. Kruskal-Wallis test with Dunn’s multiple comparisons test; significant differences are indicated in the graphs and compared to the untreated group (*, p ≤ 0.05; **, p ≤ 0.01; ***, p ≤ 0.001). (TIF) [file pone.0314973.s004.tif]

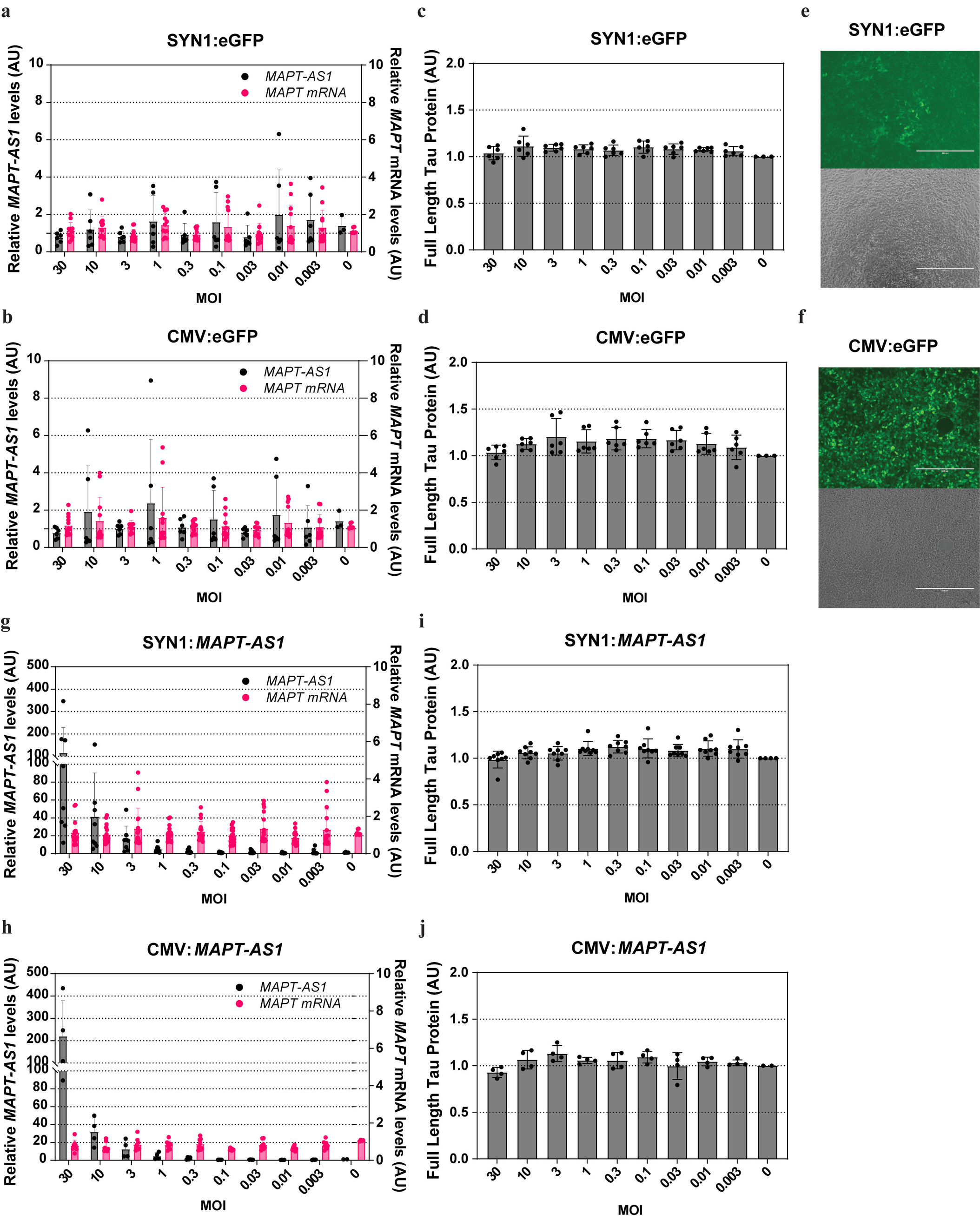

Supplement: S5 Fig — (a-j) SK-N-MC cells were treated with lentiviral constructs overexpressing (a-f) eGFP or (g-j) MAPT-AS1 and harvested 48 hours later for RNA and TAU protein analysis; n = 2–4 independent experiments. (e, f) eGFP expression was confirmed 48 hours after treatment with both SYN1:eGFP and CMV:eGFP constructs. Representative images 48 hours after treatment at MOI30; scale bar 400 μm. (a, b, g, h) RNA expression levels were evaluated by RT-sqPCR; relative RNA level values are normalized to 2 endogenous control genes and calibrated to untreated condition; data are mean ± SD. (c, d, i, j) TAU protein levels were assessed using a full-length Tau protein MSD assay; values are scaled to untreated condition (average set to 1); all data are mean ± SD. (TIF) [file pone.0314973.s005.tif]

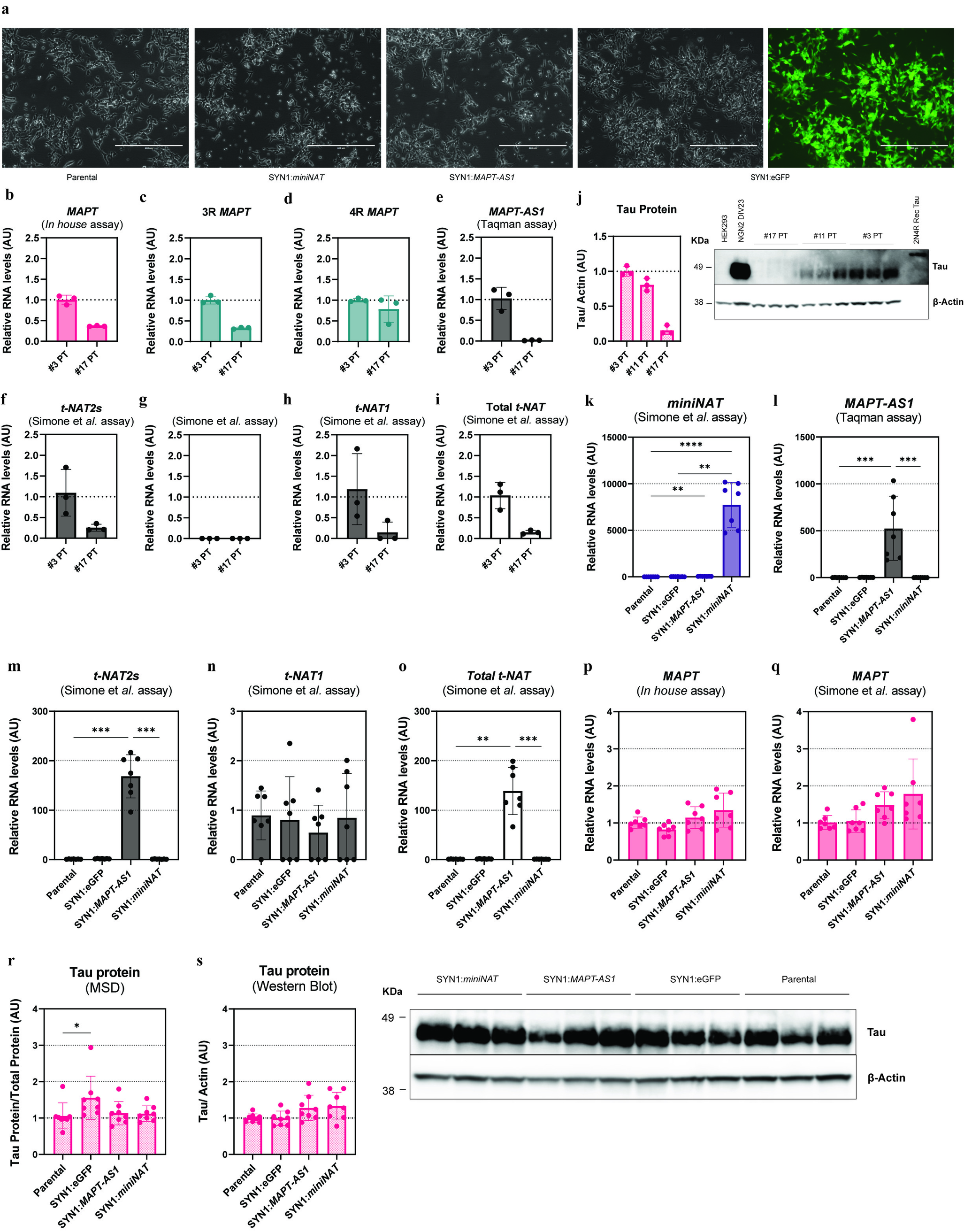

Supplement: S6 Fig — a, eGFP expression in SH-SY5Y cells was confirmed after treatment with a SYN1:eGFP construct at a multiplicity of infection (MOI) of 30. Representative images 72 hours after puromycin treatment. Scale bar 400μm. b-i, Expression levels of MAPT (b-d) or MAPT-AS1 (e) and t-NAT (f-i) transcripts in SH-SY5Y cells at passage 3 or 17 post-thawing (#3 or #17 PT, respectively). RNA expression levels were evaluated by RT-qPCR; n = 3 SH-SY5Y lysates per passage; relative RNA level values are normalized to 2 endogenous control genes and calibrated to lowest passage number (#3 PT); data are mean ± SD. j, Tau protein levels in SH-SY5Y cells at passage 3, 11 or 17 post-thawing (#3, #11 or #17 PT, respectively) were assessed using Western Blot analysis; n = 3 SH-SY5Y lysates per passage; Tau protein levels were normalized to β-actin levels and scaled to the lowest passage number (#3 PT; average set to 1); data are mean ± SD. k-s, SH-SY5Y cells were treated with lentiviral constructs at a multiplicity of infection (MOI) of 30 and cells stably expressing eGFP, MAPT-AS1 or miniNAT, with exception for the parental line which remained non-treated. Cell lysates were harvested from 3 consecutive passages for RNA and Tau protein analysis. k-o, Expression levels of miniNAT (k), MAPT-AS1 (l) and t-NAT transcripts (m-o) were evaluated by RT-qPCR; n = 2–3 independent lysates per each condition and per passage; data from different experiments indicated as circles, squares or triangles, respectively; relative RNA level values are normalized to 2 endogenous control genes and calibrated to parental line group; Dunn’s multiple comparisons test (*, p ≤ 0.05; **, p ≤ 0.01; ***, p ≤ 0.001; ****, p < 0.0001). All data are mean ± SD. p, q, MAPT mRNA expression levels in SH-SY5Y stable cell lines were evaluated by RT-qPCR using two independent primer sets; n = 2–3 SH-SY5Y lysates per passage; relative RNA level values are normalized to 2 endogenous control genes and calibrated to parental line group; Dun [file pone.0314973.s006.tif]

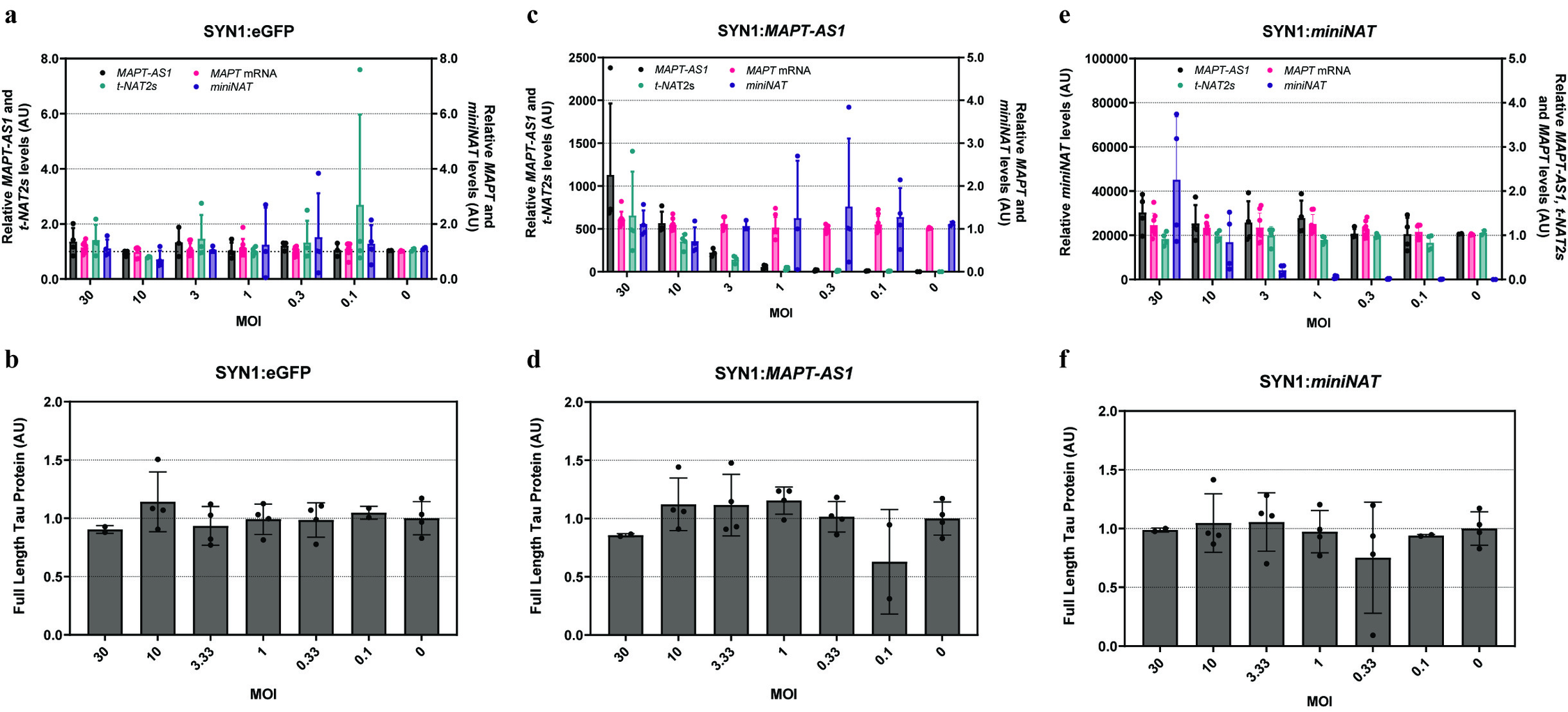

Supplement: S7 Fig — NGN2-neurons were treated with lentiviral constructs overexpressing eGFP (a, b), MAPT-AS1 (c, d) or miniNAT constructs (e, f) at day 8 and harvested 10 days later at day 18 for RNA and Tau protein analysis; n = 3 independent experiments per lentivirus. RNA expression levels were evaluated by RT-qPCR; relative RNA level values are normalized to 2 endogenous control genes and calibrated to untreated condition. Tau protein levels were assessed using a full-length Tau protein MSD assay; values scaled to untreated condition (average set to 1); all data are mean ± SD. (TIF) [file pone.0314973.s007.tif]
