# Supplementary material for: The MIR-NAT MAPT-AS1 does not regulate Tau expression in human neurons
Source: PLoS One. 2025 Jan 6;20(1):e0314973. doi: 10.1371/journal.pone.0314973 (PMC11703057; doi:10.1371/journal.pone.0314973)
Supplement: S1 File — VectorBuilder Virus ID, Abbreviation, Target RNA/Protein, Viral Type, Promotor and Titer are shown; Customized and commercially available Accel siRNAs sequences (Dharmacon) and pre-designed and custom-designed Silencer Select siRNAs (Ambion; Simone et al. publication). Full Name, Abbreviation, Reference, Sense and Antisense Sequences are shown. Abbreviation corresponds to the name given in this manuscript; ASOs sequences. ASO name, Target Gene, Sequence, Wing Chemistry and Gapmer Chemistry are shown. ASO name corresponds to the name given in this manuscript; GeNorm human reference genes, custom designed primers and IDT DNA pre-designed Taqman RT‐qPCR assays and primers. For Genorm human reference genes and custom designed primers, gene name, Primer and Sequence are shown. FWD = forward; REV = reverse; IDT DNA pre-designed Taqman RT‐qPCR assays and primers can be found at https://eu.idtdna.com/site/order/qpcr/predesignedassay. (PDF) [file pone.0314973.s008.pdf]

Table S1. Customized VectorBuilder lentiviral constructs information. VectorBuilder Virus ID, Abbreviation, Target RNA/Protein, Viral Type, Promotor and Titer are shown. Abbreviation corresponds to the name given in this manuscript.

Virus ID	Abbreviation	Target RNA/Protein	Viral type	Promoter	Titer (1st batch)	Titer (2nd batch)
**VB190716-1013rtr**	CMV:MAPT-AS1	MAPT-AS1 without polyA	Lentivirus	CMV	2.73 x 10e8tu/mL	
**VB190716-1208fkr**	SYN1:MAPT-AS1	MAPT-AS1 without polyA	Lentivirus	SYN1	4.03 x 10e8tu/mL	1.17 x 10e9tu/mL
**VB170504-1049ajc**	SYN1:eGFP	EGFP	Lentivirus	SYN1	7.5 x 10e8tu/mL	1.04 x 10e9tu/mL
**VB180802-1002wky**	CMV:eGFP	EGFP	Lentivirus	CMV	8.1 x 10e8tu/mL	
**VB210531-1081cph**	SYN1:miniNAT	miniNAT (Simone et *al.*)	Lentivirus	SYN1	4.12 x 10e8tu/mL	

Table S2. Customized and commercially available Accel siRNAs sequences (Dharmacon) and pre-designed and custom-designed Silencer Select siRNAs (Ambion; Simone et. al publication).
Full Name, Abbreviation, Reference, Sense and Antisense Sequences are shown.
Abbreviation corresponds to the name given in this manuscript.

Full Name	Abbreviation	Reference	Sense Sequence (5'-3')	Antisense Sequence (5'-3')
**Accel MAPT-AS1 s237111**	MAPT-AS1 siRNA	HR1ZN-008811	CCACUUCAUGGAUAAGUAAUU	UUACUUAUCCAUGAAGUGGUU
**Accell GAPD siRNA**	GAPDH siRNA	D-001930-01-50	AATTACTTATCCATGTTGTGG	AACCACTTCATGGATAAGTAA
**Accell Human MAPT (4137) siRNA, set of 4**	MAPT siRNA		A-012488-13, A-012488-14, A-012488-15, A-012488-16	
**Accell Non-targeting Control Pool**	Scrambled siRNA	D-001910-10-20		
**Ambion siNT1nover**	NT1 siRNA	4390828	CGGCGAGGCAGAUUUCGGAtt	UCCGAAAUCUGCCUCGCCGtc
**Ambion siNT2nover**	NT2 siRNA	4390828	GCCGCCGAGUCCGUCCACAtt	UGUGGACGGACUCGGCGGCcg
**Ambion siEx4-n268298**	Exon4_1 siRNA	4390815	GAUUUGUCAUGAGUCUCUUtt	AAGAGACUCAUGACAAAUCaa
**Ambion siEx4-n268302**	Exon4_2 siRNA	4390815	AGGACAAUGUCCUAAGGAAtt	UUCCUUAGGACAUUGUCCUcc
**Ambion Negative Control #2 siRNA***	Scrambled siRNA	4390847		

Table S3. ASOs sequences. ASO name, Target Gene, Sequence, Wing Chemistry and Gapmer Chemistry are shown. ASO name corresponds to the name given in this manuscript. MAPT-AS1 lead ASOs highlighted.

ASO name	Target Gene	Sequence (5'- 3')	Wing chemistry	Gapmer chem
**MAPT ASO**	Human MAPT	Undisclosed		
**Scrambled ASO**	Non-Targeting	CCTTCCCTGAAGGTTCCTCC	2'MOE OPS	OPS
**MALAT1 ASO**	Human MAPT-AS1	UGCCUTTAGGATTCTAGACA	2'MOE OPS	OPS
**MAPT-AS1 ASO-10**	Human MAPT-AS1	AAGATCATGTCTCTCTCTTG	2'MOE OPS	OPS
**MAPT-AS1 ASO-16**	Human MAPT-AS1	CTTTGCTGTGTCAATGTGGG	2'MOE OPS	OPS

Table S4. GeNorm human reference genes, custom designed primers and IDT DNA pre-designed Taqman RT-qPCR assays and primers.

GeNorm human reference genes; Gene name, Primer and Sequence are shown. FWD = forward; REV = reverse

Gene Name	Primer	Sequence
**GAPDH**	FWD	AAGGTGAAGGTCGGAGTCAAC
	REV	GGGGTCATTGATGGCAACAATA
**RNF20**	FWD	TTATCCCGGAAGCTAAACAGTGG
	REV	GTAGCCTCATATTCTCCTGTGC
**VIPAR**	FWD	GGGAGACCCAAAGGGGAGTAT
	REV	GGAGCGGAATCTCTCTAGTGAG
**SCLY**	FWD	ACTATAATGCAACGACTCCCCT
	REV	CTTCCTGCTGAATACGGGCTG
**PRDM4**	FWD	CACCTCCACAGTACATCCACC
	REV	TGATAGGGATCTAGTGCTGAAGG
**ENOX2**	FWD	TCATTGTGGAAGTTTTCGAGCA
	REV	TGCGGTAACCAGACAGATACA
**UBE4A**	FWD	TAGCCGCTCATTCCGATCAC
	REV	GGGATGCCATTCCCGCTTT
**UBE2DE**	FWD	CAGTCCCTATCAGGGTGGAGT
	REV	AAGGGGTAATCTGTTGGGAAATG
**ERCC6**	FWD	TCACGTCATGTACGACATCCC
	REV	GTGGCAGCTTGAGGGCTAAG

Custom designed primers; Gene name, Primer and Sequence are shown. FWD = forward; REV = reverse

Gene Name	Primer	Sequence
**MAPT (Assay 1)***	FWD	CCTCCAAGTGTGGCTCATTA
	REV	CAATCTTCGACTGGACTCTG
**MAPT (Assay 2)***	FWD	CAGTGGTCCGTACTCCA
	REV	TGGACTTGACATTCTTCAGG
**MAPT-AS1 (Assay 2)****	FWD	AGGAAGATCATGTCTCTCTCTTG
	REV	TGGAACAGTAGCCCAGGA
**MAPT-AS1 (Assay 3)****	FWD	TTCTTTGCTGTGTCAATGTG
	REV	AGTCTCTTTGTTATAGCCACT
**3R MAPT**	FWD	AGGCGGGAAGGTGCAAATA
	REV	GCCACCTCCTGGTTTATGATG
**4R MAPT**	FWD	CGGGAAGGTGCAGATAATTAA
	REV	TATTTGCACACTGCCGCCT
**MAPT (Simone et *al.*)**	FWD	GATTGGGTCCCTGGACAATA
	REV	GTGGTCTGTCTTGGCTTTGG
**Total t-NAT (Simone et *al.*)**	FWD	GGAGTCAGAACAAGGACGGG
	REV	GCACATCCTGGGCTACTGTT
**t-NAT2l (Simone et *al.*)**	FWD	CCAAGACTCCAGTTCTCGCC
	REV	CATCCTGGGCTACTGTTCCA
**t-NAT2s (Simone et *al.*)**	FWD	ACCTCCTGTCCAGGCTTCT
	REV	CCGCACACTAACTGCTTTGA
**t-NAT1 (Simone et *al.*)**	FWD	GAGGAGGAGAAGGTGGCTGT
	REV	GGACCTGGTTCCCTTCACTT
**miniNAT (Simone et *al.*)**	FWD	CGGGAGAGGTTAATACACCCA
	REV	CTGTGTCAATGTGGGCTTCT

* assays used for Fig. 2f, g (*MAPT* mRNA levels shown as average of Assays 1 and 2).

* assays used in combination with *MAPT-AS1* (Assay 1) for Supplementary Fig. 3a (*MAPT-AS1* levels shown as average of Assays 1 to 3).

IDT DNA pre-designed Taqman RT-qPCR assays and primers (Available at https://eu.idtdna.com/site/order/qpcr/predesignedassay).
Gene name, Assay ID, and Assay Configuration are shown.

Gene Name	Assay ID (IDT DNA)	Assay Configuration
**MALAT1**	Hs.PT.58.26451167.g	FAM/ZEN/IBFQ, P:P 2
**GFAP**	Hs.PT.58.1057167	DNA Primer
**TREM2**	Hs.PT.58.40294042	DNA Primer
**MAPT-AS1 (Assay 1)**	Hs.PT.58.20559679	FAM/ZEN/IBFQ, P:P 2
**MAPT (Assay 3)**	Hs.PT.58.28269192	DNA Primer
**RBFOX3**	Hs.PT.58.2776427	FAM/ZEN/IBFQ, P:P 2
**TUBB3**	Hs.PT.58.20385221	DNA Primer

* used for Supplementary Fig. 2a
