# Supplementary material for: The MIR-NAT MAPT-AS1 does not regulate Tau expression in human neurons
Source: PLoS One. 2025 Jan 6;20(1):e0314973. doi: 10.1371/journal.pone.0314973 (PMC11703057; doi:10.1371/journal.pone.0314973)
Supplement: Attachment — Submitted filename: Response to Reviewers.docx [file pone.0314973.s009.docx]

**Response to Reviewers**

We would like to thank both reviewers for taking the time to provide constructive feedback to the manuscript. Responses to each point can be found below.

**Reviewer 1**

1. Based on the data shown in Figure 1 it is not clear to me why the authors state that they don’t find a robust expression of MAPT-AS1 transcript variants t-NAT1 and t-NAT2l (line 116).

On **Fig. 1a** we include a schematic figure from all transcripts with the purpose of showing the transcripts that have been previously reported by Simone and colleagues (<https://doi.org/10.1038/s41586-021-03556-6>). This does not imply that the same transcripts have been identified or detected in our study, which mainly focuses on the *t-NAT2s* canonical isoform (NR_024559.1). For instance, our RNA-seq data detected a very small amount of reads mapping to *MAPT-AS1* isoforms besides the canonical one (see updated version of **Fig. 1**; please note that polyA-seq coverage is the equivalent to read depth). This is in line with long-read RNA seq data from the T2T consortium which does not support expression of *t-NAT2l* (see below; source: [UCSC Genome Browser Home](https://genome-euro.ucsc.edu/)). We have now also added **Table 1** to the manuscript, in order to clarify the different isoforms identified in different studies and/or databases and provide clarity on the denomination of the different transcripts. Additionally, we show (**Supplementary Fig. 1**) the Raw Ct values and normalized RNA quantities for all *t-NAT* transcripts obtained by RT-qPCR data generated from human brain samples and confirm that the Raw Ct values obtained for *t-NAT1* are higher (i.e. expressed at lower levels) compared to *t-NAT2s* and not expressed in all samples; while *t-NAT2l* is not detected at all in our cohort, confirming a lack of robustness in the detection of these transcripts, especially *t-NAT2l*. Finally, as mentioned in our Discussion section, we used a probe-based and exon-spanning assay to detect levels of the Ref Seq *MAPT-AS1* isoform, in contrast to the previously published sequences from Simone and colleagues’ study, where non-exon spanning primers were used, highlighting the robustness of our RT-qPCR data.

2. What is the authors’ consideration on the potential degradation of RNA that could occur in the human brain samples based on the post-mortem interval (qPCR results of Figure 2d-g).

We recognize that factors such as PMI and overall quality of the tissue might affect detection of some targets and this is a limitation of working with human brain samples. We appreciate the remark and checked our cohort (see below; data analysed with unpaired t-test), which does show on average a longer PMI reported in the control cases compared to patient samples. In a previously published simulation study (<https://pubmed.ncbi.nlm.nih.gov/24836634/>), the authors indicate that longer PMIs may lead to a downregulation of neuronal gene expression and gain of astroglial/microglial gene expression. However, and in contrast to the previously mentioned study, we still observe downregulation of specific neuronal targets (such as *MAPT*, *MAPT-AS1*, *RBFOX3* and *TUBB3*) and an upregulation of neuroinflammatory markers (e.g. GFAP) in patient samples, which on average display lower PMIs, suggesting that this interval is not the main driver of expression. Additionally, our results are in line with previously published data showing an overall decrease in neuronal target expression and an increase in the expression of neuroinflammatory markers in the course of AD (<http://swaruplab.bio.uci.edu:3838/bulkRNA/>; <https://doi.org/10.1038/s41586-021-03556-6>; <https://doi.org/10.1038/s41586-021-03556-6>), further increasing our confidence in the observed expression changes between patients and controls.

3. Along the text, I found the use of the term “iNeurons” (to denominate human iPSC-derived neurons) not appropriate. To the best of my knowledge, “iNeurons” refers to neurons obtained after direct conversion of fibroblast, without the need to reprogram the cells into a pluripotent state as is done in iPSC-derived neurons (see Mertens et al., 2021, Cell Stem Cell). In my opinion, the use of the term “iNeurons” in the text is not correct and could lead to misinterpretations by readers.

We agree with the reviewer and have replaced the term “iNeurons” by “NGN2-neurons” throughout the text, figures and figure legends to improve clarity.

4. Based on the data shown in Supplementary Fig 2a, RNA expression levels of MAPT-AS1 and MAPT are almost undetectable in the neuroblastoma cell lines, in particular in the SH-SY5Y cell line. However, the authors used these models to explore the functional relationship between *MAPT-AS1* and *MAPT* by modulating *MAPT-AS1* RNA levels by ASOs (in SK-N-MC cells; Supplementary Fig. 3) or by siRNAs (in SH-SY5Y cells; Supplementary Fig. 4). If *MAPT-AS1* RNA levels are too low/almost undetectable, it is possible that this gene is actually not functional in the two models, so decreasing its RNA levels (either by ASOs or siRNAs) will have no expected impact (as it was observed).

This figure shows the relative RNA quantities normalized to the average of all samples, so the fact that in SH-SY5Y and SK-N-MC these values are much lower does not mean that there is no expression, but rather that the expression is much lower compared to our human iPSC neuronal models. The following raw Ct values were obtained for these two cells lines when calculating the relative expression of *MAPT-AS1* in the experiment shown in **Supplementary Fig. 2a**: SK-N-MC: 30.5; SH-SY5Y: 34.3.

As part of our standard operating procedures to measure the levels of *MAPT-AS1* in cellular models, we include the following control conditions: (1)	“no template control” : water instead of real sample added to the mix prior to cDNA conversion and (2), “no reverse transcriptase” control i.e. the same sample was analysed without undergoing cDNA conversion as no reverse transcriptase was added in the mix, and (3) our assays are probe-based exon-spanning. These control conditions and probes ensure that the signals	are specific. We will add this additional information in our Material & Methods section – see “DNA conversion and Real-Time quantitative PCR (RT-qPCR)”. *MAPT-AS1* was not detected in these conditions for any of the cellular models analysed, further demonstrating the specificity of the signal despite the low levels of expression.

This is in line with the observation that many lncRNAs exert their functional role in a sub-stoichiometric manner, which is the mechanism purposed by Simone and colleagues (<https://doi.org/10.1038/s41586-021-03556-6>). Additionally, choosing SH-SY5Y and SK-N-MC cells as a model to evaluate the role of *MAPT-AS1* on Tau expression was a strategic approach to circumvent a potential lack of functionality, as these cell lines have been previously used in published studies that also assessed the role of *MAPT-AS1* on Tau expression (<https://doi.org/10.1038/s41586-021-03556-6>; <https://doi.org/10.1371/journal.pone.0157924>). Of note, in Supplementary Fig. 4, we transfected SH-SY5Y cells with the same *t-NAT* targeting siRNAs and using the same transfection protocol as previously published (<https://doi.org/10.1038/s41586-021-03556-6>). The siRNAs did not lead to robust changes in *t-NAT* expression, while our siRNAs in the same cells did lead to reduced expression of the intended targets.

Nonetheless, we used the complementary strategy and overexpressed *MAPT-AS1* in SK-N-MC cells and still did not see an impact on Tau protein levels using this approach (**Supplementary Fig. 5**). Lastly, in an attempt to reproduce previous findings, we also generated SH-SY5Y cells stably overexpressing a “miniNAT” construct, which has been previously described by Simone and colleagues in as the minimally required sequence to reduce Tau protein translation (<https://doi.org/10.1038/s41586-021-03556-6>). We included SH-SY5Y cells stably overexpressing eGFP and *MAPT-AS1* as negative and positive control, respectively, and noted that overexpressing miniNAT or *MAPT-AS1* also did not impact Tau protein levels. These experiments will now be included in the revised version in **Supplementary Fig. 6**. We also tested the “miniNAT” construct in iPSC derived neurons, and the results will now be included as **Supplementary Fig. 7**.

In conclusion, using the exact same tools and cellular models as reported in the literature, we found no evidence to support a role of *MAPT-AS1* in regulating Tau expression *in vitro*.

5. In Supplementary Figure 3f you can distinguish two populations of data. For example, are all the data points with the highest tau protein abundance at each ASO concentration coming from the same replicate? Would more replicates help on data interpretation?

We performed our experiments using both distinct biological and technical replicates, using proliferative cells in a 96-well plate format. Thus, even though the same amount of cells was seeded per well in distinct experiments, there is still an intrinsic variability to these cellular models. While we acknowledge that there is variability between the experiments and that having more replicates might be helpful to get robust conclusions, our work provides sufficient data and approaches showing a general lack of effect of modulating *MAPT-AS1* levels on Tau expression.

6. The authors described the impact of reducing MAPT-AS1 RNA levels on tau mRNA and protein levels in SH-SY5Y cells (lines 235-254) but Supp. Fig. 4 only shows quantification of RNA levels. Tau protein level quantification is needed to rule out the hypothesis that decreasing MAPT-AS1 RNA levels can increase tau protein abundance by increasing tau translational rate (as postulated by Simone et al.).

We agree with the reviewer and have adjusted the text accordingly . Given that there was no robust decrease in the levels of *t-NAT* transcripts in SH-SY5Y using the siRNAs previously published by Simone and colleagues, and the fact that we had already used an ASO-based approach in SK-N-MC cells which did not show changes in Tau expression upon *MAPT-AS1* KD, we decided to not evaluate Tau protein levels in this model. Thus, we specifically assessed the levels of Tau protein using ASOs in SK-N-MC cells and both ASOs and siRNAs in iNeurons, as in these experiments we observed a clear KD of *MAPT-AS1*. As stated above in reply to point 4., we have included additional data (see **Supplementary Fig. 6 and 7**) from complementary approaches that strongly support our conclusion that *MAPT-AS1* does not regulate Tau expression in our models.

7. Although the authors raised the point about the incubation time of ASOs and half-life time tau (lines 208-209), and this aspect looks to be solved for iPSC-derived neurons in Fig. 3 (MAPT ASOs and siRNAs cause a decrease in tau protein levels -Fig. 3f and 3n-), this is apparently not contemplated when applying ASOs over SK-N-MC cells (Supp. Fig. 3) or siRNAs over SH-SY5Y cells (Supp. Fig. 4). The authors choose a 10 days incubation time for ASOs and siRNAs over iPSC-derived neurons, but they used only 48h of incubation over neuroblastoma cell lines (lines 482-483). Unless a piece of data shows that 48h is enough to induce a decrease in tau protein levels in the neuroblastoma cell lines by ASOs or siRNAs, two days could be a short period of time (especially if compared versus the 10 days used on the iPSC-derived neurons).

Unfortunately, and even though we acknowledge that the incubation times in cell lines might not be enough to affect Tau protein levels, the high levels of proliferation from these models prevent us from testing prolonged incubation times. We mostly used immortalized cell lines to either validate our tools in a time-effective manner, or in an attempt to replicate previous findings. The fact that previous studies (<https://doi.org/10.1038/s41586-021-03556-6>; <https://doi.org/10.1371/journal.pone.0157924>); <https://doi.org/10.1186/s13578-018-0207-5>) have found contradictory results between distinct lines indicate that additional validation in other models is crucial to validate these mechanisms, which is the reason why we focused our efforts on human iPSC-derived neurons.

Finally, I would like to explain that I based my negative answer to question #3 (about the availability of data underlying the findings in the manuscript) on the fact that there are some claims in the manuscript based on “data not shown”.

We have included the relevant data in the revised version of the manuscript.

**Reviewer 2**

1. The authors integrated multiple RNA-seq datasets in Fig 1c/d. Why didn’t they provide an overview or a comprehensive IGV plot showing the read coverage across the entire MAPT and MAPT-AS1 regions, instead of displaying only a partial view?

The rationale to include only the regions of interest is related to the fact that the entire region *(MAPT* + *MAPT-AS1*) spans almost 250 kb, the majority intron. We zoomed in on the relevant regions to be able to properly visualize the exon coverage. The coverage would hardly be visible when displaying the entire region as the exons only represent a tiny fraction of that region. However, please check the updated version of Figure 1 (namely **Fig. 1c**) now showing full plot covering the *MAPT-AS1* region and zoomed in coverage of the identified isoforms.

2. Did the authors use an endogenous control, such as GAPDH or 18S rRNA, for the qPCR experiments shown in Fig 2d-g? If so, it should be clearly stated in the main text. Additionally, what does the "Relative RNA level" on the Y-axis represent? Given that the data range appears quite narrow, would presenting it on a "log10" scale make the differences more apparent?

As part of our standard operating procedures - please see Material and Methods section under “DNA conversion and Real-Time quantitative PCR (RT-qPCR)”: *“For each experiment,* *a panel of 8 different reference genes available in Supplementary Table S4 was evaluated, and their stability was determined using GeNorm analysis. Target gene expression levels were normalized to the 2 most stable reference genes and calibrated to a control sample/group unless specified otherwise. All expression levels are shown as calibrated normalized relative quantities (CNRQ) unless specified otherwise. Information on custom designed primers and IDT DNA pre-designed Taqman RT‐qPCR assays and primers is available at Supplementary Table S4.”*. Thus, relative RNA levels reflect CNRQ values, which are normalized to endogenous controls and calibrated to a specific sample/ group of samples – in most of our experiments, this corresponds to the Untreated group. For each experimental set up we ran all 8 reference genes and normalized the data to the two most stable ones. We updated main figure legends and the Materials and Methods text to make it more clear.

3. Could the authors provide a more detailed explanation of the 3R and 4R expression levels, as well as the changes in their ratios, as shown in Fig 2k and 2l?

4R Tau isoforms are associated with more mature forms of Tau protein, suggesting that the increased levels of this isoform in our neuronal cultures indicate a higher degree of maturity. In contrast, 3R Tau mRNA levels appear to plateau earlier in neuronal development (<https://doi.org/10.1002/alz.13784>). The presence of 3R Tau isoforms could indicate a population of both immature and mature neurons in culture. Therefore, as neuronal maturity progresses, we expect 4R Tau levels to be upregulated relative to 3R Tau, which is consistent with the observations shown in this figure. We added some more explanation in the main text.

4. The data variation in Fig 3a-g appears to be quite substantial. Could the authors provide an explanation for this variability?

We performed our experiments using both distinct biological and technical replicates, using human iPSC-derived neurons in a 96-well plate format. Thus, even though the same amount of cells was seeded per well in distinct experiments, this variability reflects results coming from distinct differentiations/ cultures. Another consideration is the fact that during culturing sometimes evaporation can occur in some wells more than others, which might affect the normalized values of expression (which are calculated relative to the untreated conditions). Finally, we would like to mention that we performed 3 or 4 independent experiments for each condition (reflected by the n number shown in the figure legends), but datapoints show technical replicates included within each independent experiment. Thus, given that we did not average our technical replicates to obtain 1 biological replicate, this data will also reflect variability within each experiment, which can increase data variability.
